# Prime-boost vaccination strategy enhances immunogenicity compared to single pneumococcal conjugate vaccination in patients receiving conventional DMARDs, to some extent in abatacept but not in rituximab-treated patients

**DOI:** 10.1186/s13075-020-2124-3

**Published:** 2020-02-22

**Authors:** Per Nived, Göran Jönsson, Bo Settergren, Jon Einarsson, Tor Olofsson, Charlotte Sværke Jørgensen, Lillemor Skattum, Meliha C. Kapetanovic

**Affiliations:** 1 Department of Infectious Diseases, Central Hospital Kristianstad, J A Hedlunds väg 5, SE-291 85 Kristianstad, Sweden; 2 Department of Clinical Sciences, Lund, Section for Rheumatology, Lund University, Lund and Skåne University Hospital, Lund, Sweden; 3 Department of Clinical Sciences Lund, Section of Infectious Diseases, Lund University and Skåne University Hospital, Lund, Sweden; 4 Department of Microbiological Diagnostics & Virology, Statens Serum Institut, Copenhagen, Denmark; 5 Department of Laboratory Medicine, Section of Microbiology, Immunology and Glycobiology, Lund, University, Lund, and Clinical Immunology and Transfusion Medicine, Region Skåne, Lund, Sweden

**Keywords:** Pneumococcal conjugate vaccine, Rituximab, Abatacept, Synthetic disease-modifying antirheumatic drugs

## Abstract

**Objective:**

To explore whether a prime-boost vaccination strategy, i.e., a dose of pneumococcal conjugate vaccine (PCV) and a dose of 23-valent polysaccharide vaccine (PPV23), enhances antibody response compared to single PCV dose in patients with inflammatory rheumatic diseases treated with different immunosuppressive drugs and controls.

**Methods:**

Patients receiving rituximab (*n* = 30), abatacept (*n* = 23), monotherapy with conventional disease-modifying antirheumatic drugs (cDMARDs, methotrexate/azathioprine/mycophenolate mofetil, *n* = 27), and controls (*n* = 28) were immunized with a dose PCV followed by PPV23 after ≥ 8 weeks. Specific antibodies to 12 serotypes included in both vaccines were determined using a multiplex microsphere immunoassay in blood samples before and 4–8 weeks after each vaccination. Positive antibody response was defined as ≥ 2-fold increase from pre- to postvaccination serotype-specific IgG concentration and putative protective level as IgG ≥ 1.3 μg/mL. The number of serotypes with positive antibody response and IgG ≥ 1.3 μg/mL, respectively, after PCV and PCV + PPV23 were compared within each treatment group and to controls. Opsonophagocytic activity (OPA) assay was performed for serotypes 6B and 23F.

**Results:**

Compared to single-dose PCV, prime-boost vaccination increased the number of serotypes with positive antibody response in patients with abatacept, cDMARDs, and controls (*p* = 0.02, *p* = 0.01, and *p* = 0.01), but not in patients on rituximab. After PCV + PPV23, the number of serotypes with positive antibody response was significantly lower in all treatment groups compared to controls but lowest in rituximab, followed by the abatacept and cDMARD group (*p* < 0.001). Compared to PCV alone, the number of serotypes with putative protective levels after PCV + PPV23 increased significantly only in patients in cDMARDs (*p* = 0.03) and controls (*p* = 0.001). Rituximab treatment was associated with large reduction (coefficient − 8.6, *p* < 0.001) and abatacept or cDMARD with moderate reductions (coefficients − 1.9 and − 1.8, *p* = 0.005, and *p* < 0.001) in the number of serotypes with positive antibody response to PCV + PPV23 (multivariate linear regression model). OPA was reduced in rituximab (Pn6B and Pn23F, *p* < 0.001), abatacept (Pn23F, *p* = 0.02), and cDMARD groups (Pn6B, *p* = 0.02) compared to controls.

**Conclusions:**

Prime-boost strategy enhances immunogenicity compared to single pneumococcal conjugate vaccination in patients with inflammatory rheumatic diseases receiving cDMARDs, to some extent in abatacept but not in patients on rituximab. Pneumococcal vaccination should be encouraged before the initiation of treatment with rituximab.

**Trial registration:**

ClinicalTrials.gov, NCT03762824. Registered on 4 December 2018, retrospectively registered

## Key messages

1. Prime-boost vaccination is more immunogenic than single pneumococcal vaccine in cDMARD or abatacept-treated patients.
2. Compared to single vaccine, prime-boost pneumococcal vaccination strategy is not more immunogenic in rituximab-treated patients.
3. Pneumococcal vaccination should ideally be performed prior to initiation of rituximab treatment.

## Introduction

Infection, in particular pneumonia, is an important cause of the excess mortality in patients with rheumatoid arthritis [1]. Invasive pneumococcal disease (IPD), caused by *Streptococcus pneumoniae*, is a vaccine-preventable life-threatening condition. In a 2018 meta-analysis, the pooled incidence of IPD was 65/100,000 person years in patients with chronic inflammatory disease, compared to 10/100,000 in healthy controls [2].

Two pneumococcal vaccines are currently available for immunization of adults, the 23-valent pneumococcal polysaccharide (PPV23) and the 13-valent conjugate vaccine (PCV13). Twelve serotypes are common to both vaccines and PPV23 includes 11 additional serotypes. In contrast to PPV23, the conjugate vaccines induce a T cell-dependent (TD) immune response, resulting in the production of memory B cells. The introduction of 7-valent pneumococcal conjugate vaccine (PCV7) followed by PCV13 into childhood vaccination programs worldwide resulted in herd protective effects [3] and shifts toward IPD caused by non-PCV13 serotypes, i.e., serotype replacement has been reported from several countries [4–6]. A large randomized placebo-controlled trial of adults ≥ 65 years demonstrated that PCV13 effectively prevented vaccine-type IPD and pneumococcal pneumonia in 75% and 45%, respectively [7]. However, among all IPD cases in Sweden in 2017, PCV13-serotypes accounted for only 30%, but 65% of infections were caused by serotypes included in PPV23 [8]. Since 2012, the Centers for Disease Control and Prevention (CDC) Advisory Committee on Immunization Practices (ACIP) recommendations for adults with immunocompromising conditions is to receive immunization with a dose of PCV13, followed after at least 8 weeks by a dose of PPV23, because of the wider serotype coverage [9]. The European Society of Clinical Microbiology and Infectious Diseases (ESCMID) Vaccine Study Group (EVASG) also recommends that at-risk adults receive this vaccine schedule [10], which we refer to as the prime-boost pneumococcal vaccination strategy [11]. The European League Against Rheumatism (EULAR) strongly recommends pneumococcal vaccination in adult patients with autoimmune inflammatory rheumatic disease, but does not provide a specific recommendation due to the lack of evidence on the efficacy, immunogenicity, and safety of available pneumococcal vaccines [12].

A study of 24 RA patients who received prime-boost vaccination did not find significant differences in percentages of protective antibodies between the common and uncommon vaccine serotypes, and functional antibodies did not persist for 2 years [13]. Moreover, Nguyen et al. found no significant differences in initial antibody response between single- or double-dose PCV13 followed by PPV23 in RA patients treated with biologics compared to prime-boost vaccination in a group treated with conventional DMARDs [14]. Whether the prime-boost strategy has advantages over single-dose PCV13 or PPV23 in patients with inflammatory rheumatic diseases, and in the context of different immunosuppressive treatments are still not fully understood. The aim of this study was to investigate if the combined schedule of PCV13 followed by PPV23 improved antibody response compared with single-dose PCV13 in patients with inflammatory rheumatic diseases (IRD) during various immunosuppressive therapies and in healthy controls.

## Materials and methods

### Patient inclusion

Adult patients with inflammatory rheumatic disease, regularly monitored at the Departments of Rheumatology at Skåne University Hospital in Lund and Central Hospital in Kristianstad, were eligible for this study. At inclusion in the study, all patients’ medical records were scrutinized in order to confirm that patients fulfilled the American College of Rheumatology (ACR)/European League Against Rheumatism (EULAR) criteria for RA or ACR criteria for systemic vasculitides [15, 16]. Ongoing treatment at the time of vaccination was noted as a basis for later patient stratification. Patients were eligible for prime-boost pneumococcal vaccination if they had not previously received pneumococcal conjugate vaccine and they had not received PPV23 within the last 5 years. Patients previously immunized with one dose pneumococcal conjugate vaccine but no PPV23 within the VACCIMIL (Vaccination in Inflammatory Rheumatic Disease) study [17] were eligible for immunization with a PPV23 booster dose within the present study. Patients were excluded from the study if antirheumatic treatment had been changed within 4 weeks before vaccination, had a history of allergic reaction at previous vaccinations, were pregnant, or had an ongoing infection. Healthy control subjects were recruited from the staff and relatives at the Department of Rheumatology in Lund.

### Treatment groups and controls

Patients were treated with rituximab (RTX, *n* = 45), abatacept (ABT, *n* = 23), monotherapy with conventional DMARD (cDMARD, *n* = 27, i.e., methotrexate [MTX]/azathioprine [AZA]/mycophenolate mofetil [MMF]), and 28 healthy controls participated in the study. The modified RTX group (*n* = 30) consisted of patients that had been started on treatment with RTX (at least 2 doses) before receiving PCV immunization (PCV13 *n* = 20; PCV7 *n* = 10), and they had ongoing RTX at the time of PPV23 immunization. In the RTX group (*n* = 30), 16 patients (53%) had concomitant treatment with MTX. In addition, 15 patients (the RTX-PPV23 group) who had previously received a PCV dose before the start of RTX treatment were immunized with a PPV23 booster dose and were analyzed separately.

**Fig. 1:**
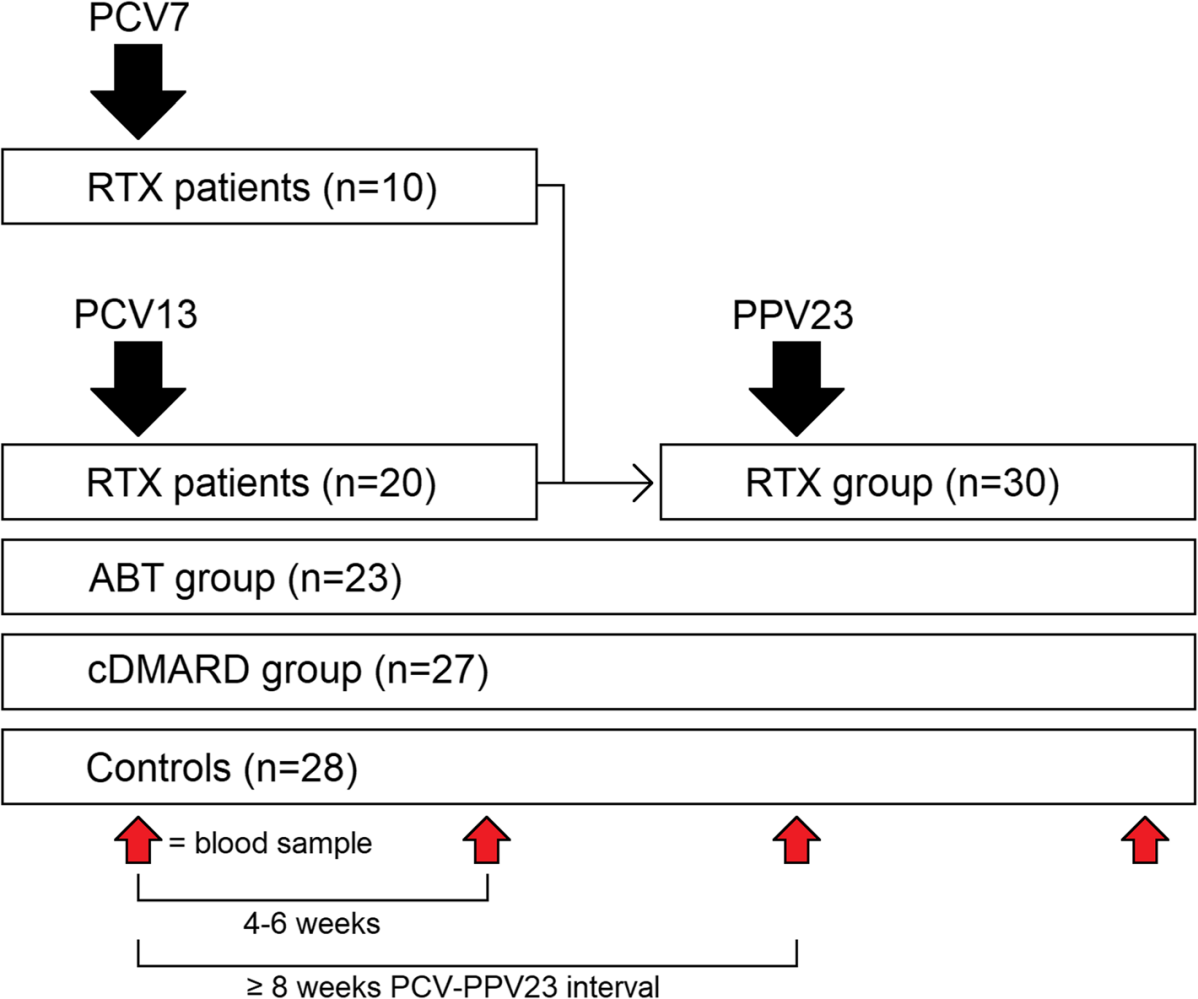
Schematic of PCV and PPV23 immunizations and blood samples in treatment groups and controls

Patients and controls were included in this study in accordance with Fig. 1. Demographic data, diagnoses, disease characteristics, and medication details in the main treatment groups RTX (*n* = 30), ABT (*n* = 23), cDMARD (*n* = 27), and in controls (*n* = 28) are summarized in Table 1. In the additional RTX-PPV23 group (*n* = 15, median age 71 years, 73% female), patients were diagnosed with RA (*n* = 8), granulomatosis with polyangiitis (GPA, *n* = 4), eosinophilic GPA (*n* = 1), and other systemic vasculitis (*n* = 2). In this group, median RTX treatment duration was 2.1 years (range 0.3–6.9 years) and concomitant treatments were MTX/AZA/MMF (*n* = 6, median duration 9.5 years [2.7–16.7 years]) and prednisolone (*n* = 12; median dose, 5 mg [0–15 mg]).

**Table 1:** Demographic, diagnoses, disease characteristics, and treatment at inclusion in the study, in treatment groups and controls

	Rituximab	Abatacept	cDMARD^1^	Controls
*N*	30	23	27	28
Female gender, %	53%	83%	74%	64%
Age, median (range) years	69 (31–88)^2^	64 (42–78)^2^	68 (25–87)^2^	55 (18–84)
Rheumatoid arthritis, *n* (%)	27 (90%)	23 (100%)	14 (52%)	0
RF-positive (% of RA patients)	100%	79%	90%	–
Anti-CCP-positive (% of RA patients)	92%	68%	80%	–
Granulomatosis with polyangiitis, *n* (%)	3 (10%)	0	7 (26%)	0
Eosinophilic granulomatosis with polyangiitis, *n* (%)	0	0	3 (11%)	0
Other systemic vasculitis, *n* (%)	0	0	3 (11%)	0
Disease duration, median (range) years	20 (2–57)	15 (4–45)	5 (2–46)	–
DAS28 in RA patients, median (range)	2.7 (0.5–6.5)^3^	3.2 (1.5–5.5)	2.3 (1.6–4.3)	–
CRP, median mg/L	3.0	2.3	3.1	0.7
Total IgG, median (range) g/L	7.4 (4.0–13.9)	–	–	–
RTX duration, median (range) years	6.3 (0.7–10.9)	–	–	–
ABT duration, median (range) years	–	3.7 (0.7–10.2)	–	–
cDMARD duration, median (range) years	12.9 (3.3–22.4)	10.3 (4.2–20.3)	3.5 (1.4–15.3)	–
MTX, *n* (%)	16 (53%)	11 (48%)	19 (70%)	0
MTX mg/week, median	15	20	20	0
Azathioprine, *n* (%)	1 (3%)	0	5 (19%)	0
Azathioprine mg/day, median	150	0	100	0
Mycophenolate mofetil, *n* (%)	0	0	3 (11%)	0
Mycophenolate mofetil mg/day, median	0	0	1500	0
Prednisolone, *n* (%)	10 (33%)	10 (43%)	15 (56%)	0
Prednisolone mg/day, median (range)	5 (2.5–15)	5.6 (2.5–20)	5 (2.5–15)	0
Previous treatment with TNFα-inhibitor (%)	72.4	80.0	11.1^4^	0

^1^Conventional disease-modifying antirheumatic drugs: methotrexate, azathioprine, or mycophenolate mofetil

^2^All treatment groups were older than controls (all *p* < 0.05)

^3^DAS28 did not differ between treatment groups

^4^In the cDMARD group, compared to other treatment groups, a lower proportion of patients had previously received TNFα-inhibitor treatment

### Vaccination protocol

All participants fulfilling criteria for prime-boost pneumococcal vaccination were immunized with a single 0.5 mL dose of PCV13 (Prevenar 13®, Pfizer), followed after 8 weeks by a single 0.5 mL dose of PPV23 (Pneumovax®, MSD), administered as intramuscular injections in the deltoid muscle by a physician or nurse. Participants previously immunized with a single dose of PCV13 within the VACCIMIL study received a single 0.5 mL dose of PPV23 in the present study. In the RTX group (*n* = 30), a subgroup of 10 patients had previously received a single 0.5 mL dose of PCV7 (Prevenar®, Pfizer), and they were immunized with a 0.5 mL dose of PPV23. At the time of vaccination, data were collected on disease and treatment characteristics and previous vaccinations using a structured protocol. All patients were encouraged to monitor and report possible adverse or unexpected effects of the vaccination, as well as changes in rheumatic disease. Adverse events (AEs) and adverse drug reactions (ADRs) were recorded according to the Guideline for Good Clinical Practice and Clinical Safety Data Management [18].

### Serum samples

For all participants receiving prime-boost pneumococcal vaccination, serum samples were collected immediately before administration of PCV13 and PPV23 vaccines and 4–6 weeks after PPV23. For participants previously included in the VACCIMIL study, serum samples were drawn immediately before administration of PPV23 vaccine and 4–6 weeks after, and frozen serum samples taken immediately before and 4–6 weeks after prior PCV vaccination were re-analyzed.

### Multiplex fluorescent microsphere immunoassay (MFMI)

Sera were frozen at − 80 °C and subsequently analyzed at Statens Serum Institut, Copenhagen, Denmark. Pneumococcal serotype-specific IgG concentrations were determined for the 12 capsular serotypes (1, 3, 4, 5, 6B, 7F, 9 V, 14, 18C, 19A, 19F, and 23F) common to both PCV13 and PPV23, using an in-house MFMI (Luminex) based on the procedure previously described by Lal et al. [19]. This method permits simultaneous measurement of antibodies to all 12 serotypes in a single sample.

### Opsonophagocytic activity (OPA) assay

A flow-cytometric pneumococcal uptake OPA assay was performed for pneumococcal serotypes 6B and 23F, the two serotypes available at our laboratory. The method has been described by Martinez et al. [20] and was executed with some modifications; briefly, neutrophils from healthy donors were used instead of HL-60 cells and results were expressed as percentage of cells with bacterial uptake [17].

### Statistical analysis

Serotype-specific IgG concentrations were log-transformed to calculate geometric mean concentrations (GMC) with 95% confidence intervals (CI). We used Bonferroni correction to avoid type I errors in multiple serotype comparisons. Positive antibody response was defined as at least twofold increase from pre- to postvaccination serotype-specific antibody concentration. Putative protective level for each serotype was defined as specific IgG concentration ≥ 1.3 μg/mL, as recommended by the American Academy of Allergy, Asthma & Immunology [21]. Sums of serotypes with positive antibody responses and putative protective levels, respectively, were calculated for each study participant. In the PCV7-RTX subgroup (*n* = 10), only the seven serotypes included in this vaccine was evaluated. Assuming that the immune response to the PCV7-serotypes is comparable to that of the five additional serotypes in PCV13, the sum of responding serotypes was multiplied with $\frac{12}{7}$ in this subgroup. Differences between treatment groups and controls were tested using *T* test or Mann-Whitney *U* test as appropriate. Pre- to postvaccine differences within groups were tested using Wilcoxon matched-pairs signed*-*ranks test*.* We used multivariate linear regression to examine the possible influence of different exposure variables on outcome, i.e., the number of serotypes with positive antibody response. The following variables were included in a multivariate regression model: gender, age (years), C-reactive protein (CRP, mg/L), ongoing rituximab (yes/no), abatacept (yes/no), cDMARD (yes/no), and prednisolone dose (mg/day). In a stepwise selection procedure, (1) each variable was omitted and, in turn, *p* value for each likelihood ratio test was recorded, and (2) the model was fitted with all variables except for the one with highest *p* value in step one. Steps 1 and 2 were repeated until only variables with *p* < 0.10 were left to be included in the final model. All calculations were performed using R 3.5.3 software.

## Results

### Pneumococcal serotype-specific IgG concentrations

Geometric mean concentrations in treatment groups and controls are shown in Table 2.

**Table 2:** Pneumococcal serotype-specific IgG GMC (95% confidence interval [CI]) in μg/mL before vaccination, post-PCV and post-PPV23 in treatment groups and controls

	Rituximab (*n* = 30)			Abatacept (*n* = 23)			cDMARD (*n* = 27)			Controls (*n* = 28)		
Serotype	Pre-PCV	Post-PCV	Post-PPV23	Pre-PCV13	Post-PCV13	Post-PPV23	Pre-PCV13	Post-PCV13	Post-PPV23	Pre-PCV13	Post-PCV13	Post-PPV23
1	–	–	–	0.11 (0.06–0.21)	0.37 (0.19–0.73)	0.40 (0.22–0.73)	0.10 (0.05–0.18)	0.27 (0.12–0.62)	0.84 (0.40–1.77)	0.12 (0.08–0.16)	1.09 (0.58–2.03)	3.21 (1.80–5.75)
3	–	–	–	0.10 (0.05–0.19)	0.35 (0.12–1.01)	0.38 (0.15–0.98)	0.06 (0.03–0.11)	0.14 (0.07–0.25)	0.21 (0.10–0.45)	0.08 (0.04–0.16)	0.68 (0.33–1.40)	1.90 (0.83–4.36)
4	0.13 (0.07–0.23)	0.17 (0.10–0.29)	0.12 (0.07–0.21)	0.13 (0.07–0.24)	0.28 (0.14–0.58)	0.29 (0.14–0.60)	0.11 (0.07–0.17)	0.36 (0.19–0.69)	0.77 (0.29–2.02)	0.18 (0.12–0.28)	2.15 (1.09–4.26)	4.07 (1.93–8.56)
5	–	–	–	0.17 (0.07–0.43)	0.48 (0.20–1.14)	0.53 (0.23–1.24)	0.11 (0.07–0.17)	0.54 (0.23–1-27)	0.89 (0.39–2.08)	0.09 (0.06–0.13)	0.61 (0.36–1.03)	1.52 (0.77–3.00)
6B	0.16 (0.09–0.29)	0.22 (0.11–0.42)	0.17 (0.08–0.34)	0.20 (0.10–0.39)	0.77 (0.33–1.79)	0.76 (0.33–1.77)	0.17 (0.08–0.38)	0.62 (0.27–1.43)	0.67 (0.28–1.65)	0.16 (0.07–0.33)	1.32 (0.56–3.14)	2.41 (1.02–5.69)
7F	–	–	–	0.31 (0.20–0.46)	1.80 (0.90–3.60)	1.89 (0.95–3.74)	0.51 (0.29–0.91)	2.57 (1.31–5,05)	2.42 (1.25–4.68)	0.23 (0.12–0.46)	3.48 (2.02–6.02)	4.04 (2.03–8.05)
9V	0.15 (0.08–0.25)	0.17 (0.10–0.30)	0.14 (0.08–0.24)	0.22 (0.11–0.45)	0.93 (0.45–1.91)	0.92 (0.44–1.96)	0.12 (0.07–0.23)	0.58 (0.26–1.26)	0.58 (0.32–1.07)	0.15 (0.09–0.25)	1.60 (0.87–2.94)	1.94 (1.09–3.47)
14	3.15 (1.70–5.82)	3.93 (2.22–6.96)	3.24 (1.84–5.79)	1.60 (0.70–3.71)	5.89 (3.10–11.18)	6.44 (3.61–11.49)	0.54 (0.26–1.11)	2.89 (1.31–6.33)	3.28 (1.66–6.46)	1.10 (0.50–2.43)	6.77 (3.50–13.12)	13.07 (9.36–18.26)
18C	0.69 (0.43–1.10)	0.83 (0.53–1.31)	0.60 (0.36–1.00)	0.76 (0.35–1.63)	3.90 (1.97–7.72)	4.21 (2.32–7.63)	0.41 (0.24–0.71)	2.83 (1.76–4.56)	2.37 (1.42–3.95)	1.03 (0.59–1.81)	5.74 (3.91–8.41)	6.33 (4,07–9.82)
19A	–	–	–	0.31 (0.12–0.80)	1.01 (0.37–2.76)	1.05 (0.37–3.02)	0.20 (0.09–0.43)	0.86 (0.33–2.29)	0.71 (0.27–1.88)	0.20 (0.08–0.47)	1.40 (0.60–3.29)	2.19 (0.94–5.07)
19F	0.84 (0.51–1.39)	0.96 (0.58–1.60)	0.64 (0.38–1.09)	0.76 (0.39–1.46)	1.25 (0.68–2.33)	1.53 (0.74–3.14)	0.85 (0.46–1.60)	1.89 (0.83–4.32)	4.38 (1.76–10.93	0.78 (0.48–1.25)	3.09 (1.59–6.03)	12.52 (5.35–29.30)
23F	0.32 (0.20–0.53)	0.41 (0.26–0.66)	0.34 (0.21–0.55)	0.70 (0.40–1.23)	3.10 (1.50–6.36)	2.83 (1.35–5.93)	0.34 (0.19–0.62)	1.17 (0.50–2.74)	1.46 (0.73–2.94)	0.41 (0.23–0.71)	6.12 (3.23–11.61)	6.30 (3.25–12.21)

In the RTX group (*n* = 30), primary immunization with PCV13 (*n* = 20)/PCV7 (*n* = 10) only resulted in increased serotype-specific IgG concentration for 1/7 common serotypes (6B, *p =* 0.03). Combined PCV and subsequent PPV23 to patients with RTX did not increase specific IgG, compared neither to PCV alone nor to prevaccination IgG levels. Compared to controls, no differences in prevaccination serotype-specific IgG were found, but lower antibody levels were observed for 4/7 common serotypes post-PCV (all *p* < 0.001) and for 7/7 serotypes post-PPV23 (all *p* < 0.001). In the PCV13 subgroup (*n* = 20), 7/12 serotypes were reduced post-PCV13 (4/12 *p* < 0.001, 3/12 *p* < 0.01) and 11/12 post-PPV23 (8/12 *p* < 0.001, 2/12 *p* < 0.01, 1/12 *p =* 0.02), compared to controls. Compared to controls, the PCV7 subgroup (*n* = 10) showed reductions post-PCV7 for 4/7 serotypes (2/7 *p* < 0.001, 2/7 *p* < 0.01), and post-PPV23 for 7/7 common (6/7 *p* < 0.001, 1/7 *p* < 0.01) and 5/5 uncommon serotypes (3/12 *p* < 0.001, 2/12 *p* < 0.01). In the RTX-PPV23 group (*n* = 15), with previous PCV before starting RTX, a PPV23 booster dose during RTX treatment did not result in increased antibody levels. Because of the small sample size, this group was excluded from further analyses in this study.

Immunization of patients in the ABT group (*n* = 23) resulted in increased serotype-specific IgG for 9/12 serotypes (3/12 *p* < 0.001, 5/12 *p* ≤ 0.01, 1/12 *p* < 0.05) post-PCV13. Although IgG levels were not significantly increased post-PPV23 compared to post-PCV13, combined vaccination resulted in increases for 11 of the common serotypes (3/12 *p* < 0.001, 6/12 *p* < 0.01, 2/12 *p* < 0.05), compared to prevaccination antibody levels. Comparing the ABT group to controls, there were no differences before vaccination, but lower IgG were found for serotype 4 post-PCV13 (*p* < 0.001) and 3 serotypes post-PPV23 (1 and 4 *p* < 0.001, 19F *p =* 0.002).

In the cDMARD group (*n* = 27), immunization with PCV13 resulted in increased specific IgG for all serotypes (6/12 *p* < 0.001, 4/12 *p* < 0.01, 2/12 *p* ≤ 0.05). There were no differences in antibody levels post-PPV23 compared to post-PCV13. Compared to controls, no differences in specific IgG were found before vaccination but antibody levels were lower in 4/12 serotypes post-PCV13 (1/12 *p* = 0.003, 3/12 *p* < 0.05) and 7/12 serotypes post-PPV23 (1/12 *p <* 0.001, 2/12 *p <* 0.01, 4/12 *p* < 0.05).

In the control group (*n* = 28), a PCV13 dose resulted in increased specific IgG for all serotypes (all *p* < 0.001) and further increases were observed in serotypes 1, 3, 5, and 19F after PPV23 (3 and 19F *p* ≤ 0.001, 1 and 5 *p* < 0.01).

### Positive antibody responses

**Fig. 2:**
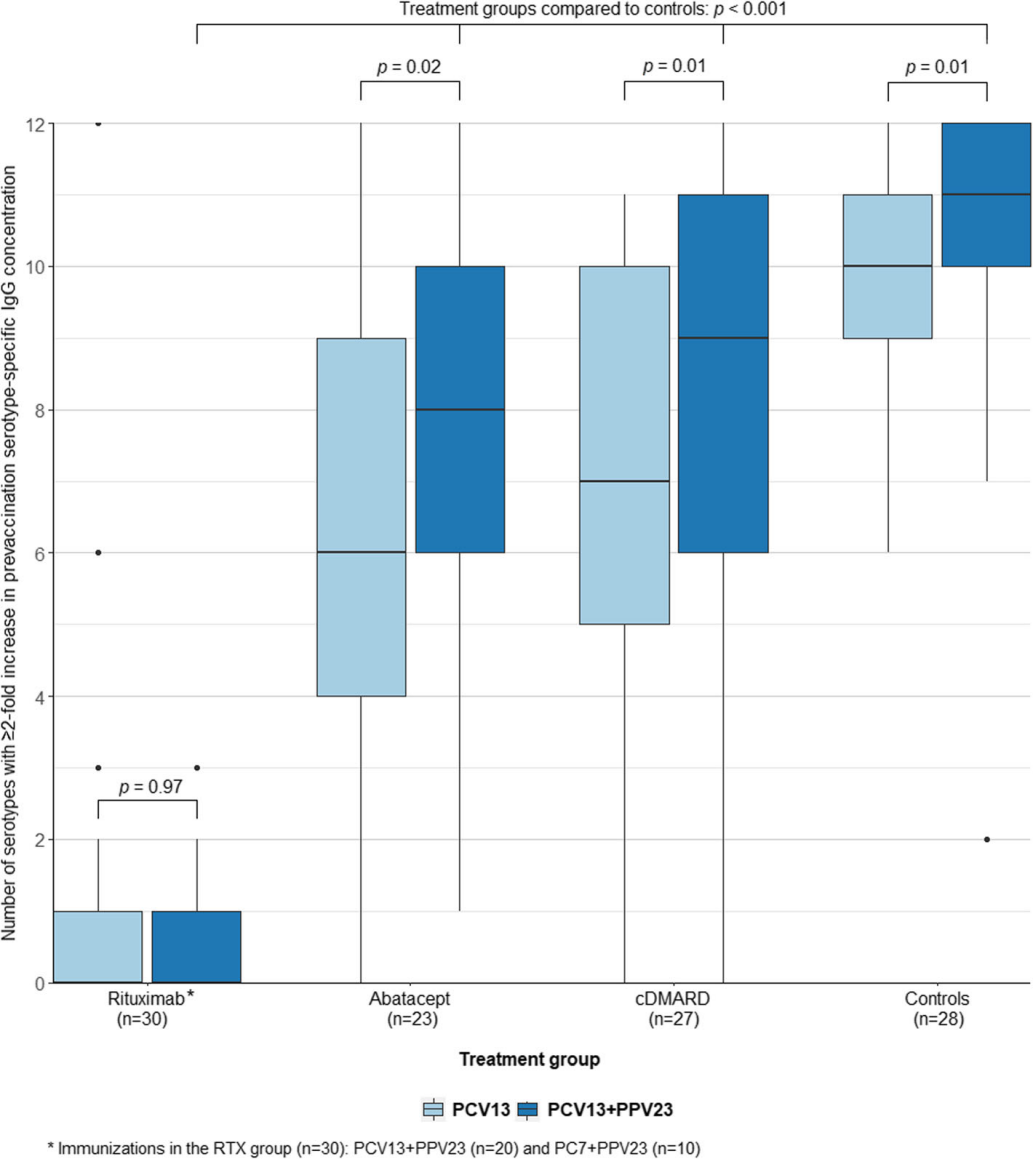
The number of serotypes with positive antibody response after PCV13 and PCV13 + PPV23 in treatment groups and controls

The numbers of serotypes with positive antibody responses (≥ 2-fold increase from prevaccination specific IgG concentration) in treatment groups and controls after PCV and PPV23 are shown in Fig. 2. PCV13 + PPV23 compared to single-dose PCV13 resulted in an increased number of serotypes with positive responses in the ABT (*p =* 0.016), cDMARD (*p =* 0.013), and control groups (*p =* 0.007). Compared to controls, the numbers of serotypes with positive response after PCV + PPV23 were reduced in all patients groups (*p* < 0.001).

### Putative protective levels

**Fig. 3:**
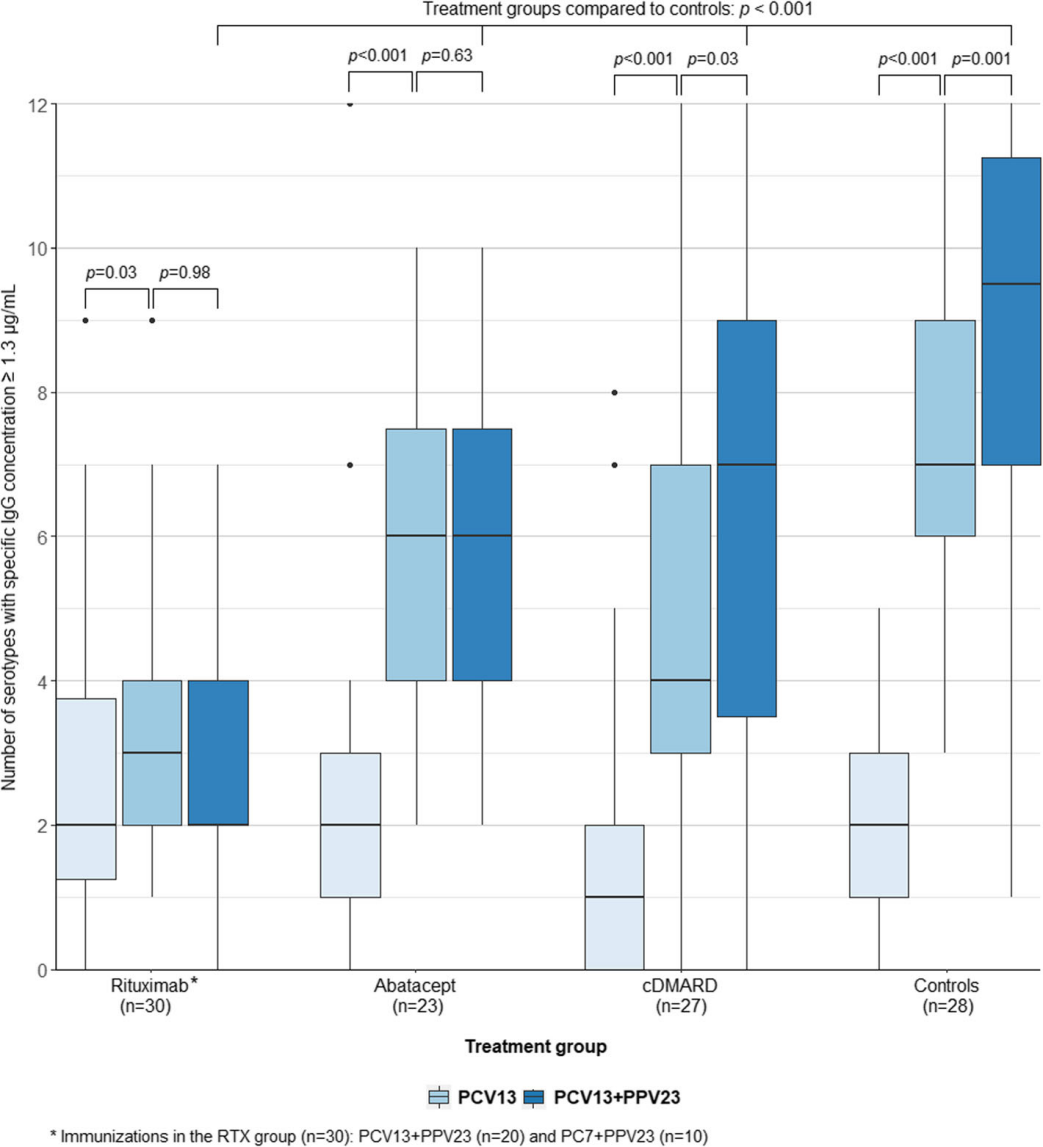
The number of serotypes with putative protective levels (i.e., specific IgG concentration ≥ 1.3 μg/mL) after PCV13 and PCV13 + PPV23 in treatment groups and controls

In the RTX group, the number of serotypes with putative protective antibody level (specific IgG concentration ≥ 1.3 μg/mL) increased slightly pre- to post-PCV (median 2 to 3, *p* = 0.03), but no further increase was seen post-PPV23 (*p* = 0.98, Fig. 3). In the ABT group, there was a pre- to post-PCV13 increase from median 2 to 6 serotypes with protective level (*p* < 0.001), but no changes were seen post-PPV23 (*p* = 0.63). The number of serotypes ≥ 1.3 μg/mL in the cDMARD group increased pre- to post-PCV13 (median 1 to 4, *p* < 0.001) and post-PPV23 (median 4 to 7, *p* = 0.03). Comparing treatment groups with controls, no significant differences in the number of serotypes with protective levels before vaccination were found. Post-PCV protective levels in the RTX, ABT, and cDMARD groups were reduced compared to controls (*p* < 0.001, *p* = 0.02, and *p* = 0.002). Post-PPV23 protective levels were reduced in all groups compared with controls (RTX, *p* < 0.001; ABT, *p* < 0.001; and cDMARD, *p* < 0.001).

### Predictors of positive antibody response to prime-boost pneumococcal vaccination

A multivariate linear regression model was derived in a stepwise selection procedure, where gender, age, CRP, and prednisolone dose were omitted each in turn because they were not associated with the outcome (likelihood test, all *p* ≥ 0.10), i.e., the number of serotypes with positive antibody response (Table 3). Rituximab was found to be an independent risk factor associated with a large reduction and abatacept and cDMARD with a moderate reduction in number of serotypes with positive antibody response (Table 3). Within the RTX group, the number of responding serotypes was not associated with rituximab treatment duration (data not shown). In a separate, multivariate regression model of positive antibody response in RA patients, DAS28 was not associated with the outcome (*p* = 0.61).

**Table 3:** Predictors of the number of serotypes (0–12) with positive antibody response, i.e., ≥ 2-fold increase from prevaccination serotype-specific [IgG], after prime-boost vaccination

	Stepwise selection of exposure variables, *p* of likelihood ratio test				Multivariate linear regression model		
Predictors:	1	2	3	4	Coefficient estimate	95% CI	*p*
Intercept (control)					**11.2**	10.3, 12.1	< 0.001
Rituximab (yes/no)	< 0.001	< 0.001	< 0.001	< 0.001	**− 8.6**	− 9.8, − 7.4	< 0.001
Abatacept (yes/no)	0.008	0.009	0.009	0.007	**− 1.9**	− 3.2, − 0.6	0.005
cDMARD (yes/no)	< 0.001	< 0.001	< 0.001	< 0.001	**− 1.8**	− 2.8, − 0.8	< 0.001
Gender	0.11	0.13	0.13	0.10	Goodness of fit: multiple *R*^2^ = 0.69		
Age (years)	0.59	–	–	–			
CRP (mg/L)	0.38	0.39	0.36	–			
Prednisolone dose (mg/day)	0.56	0.63	–	–			

### Opsonophagocytosis of pneumococcal serotypes 6B and 23F

**Fig. 4:**
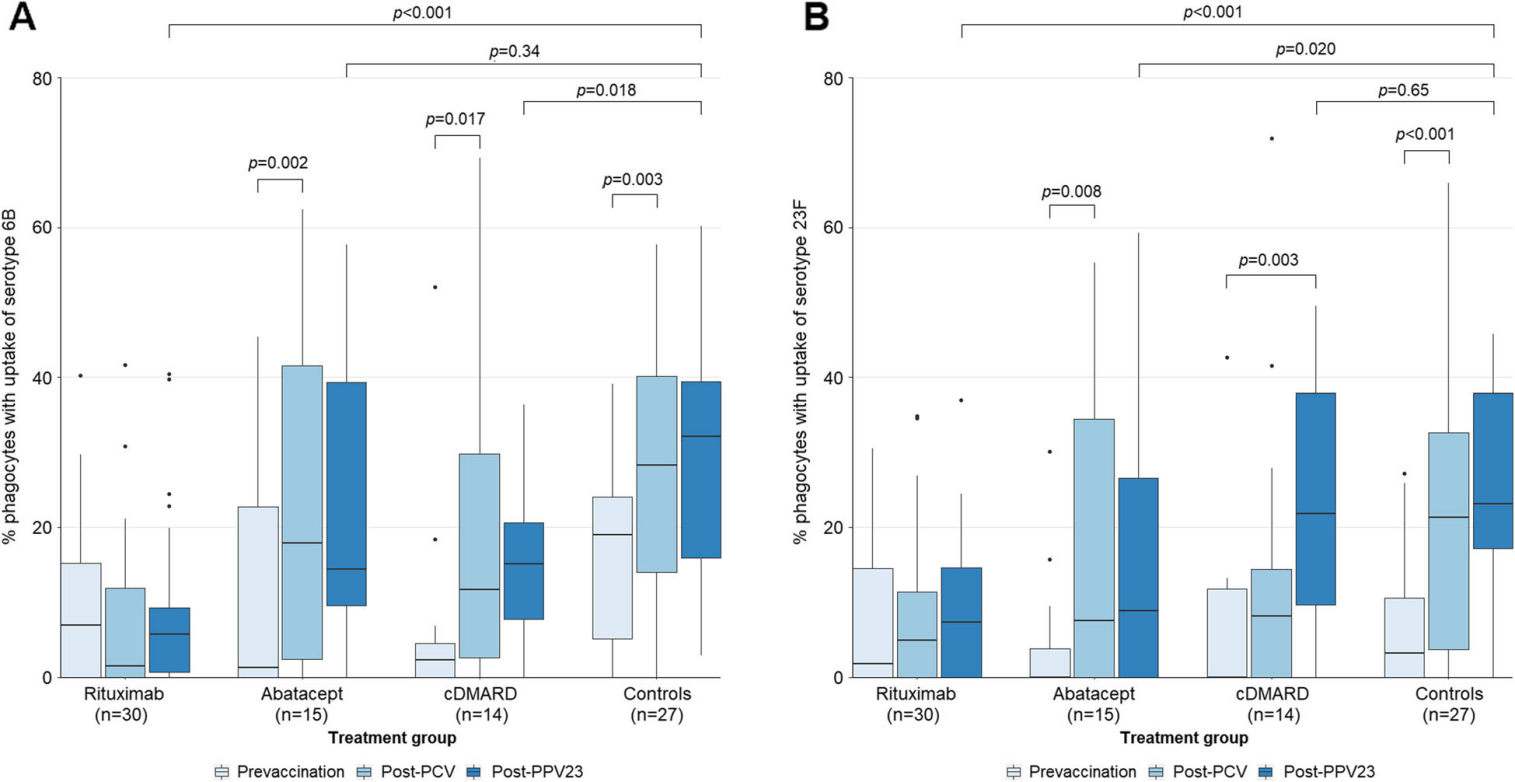
Proportion of phagocytes with uptake of pneumococcal serotype 6B (**a**) and serotype 23F (**b**)

In the rituximab group, functionality of antibodies for pneumococcal serotypes 6B (Pn6B) and 23F (Pn23F), as measured by OPA assay, neither increased after PCV prime nor PPV23 boost immunization, and post-PPV23 OPA was reduced compared to controls (both serotypes *p* < 0.001, Fig. 4). In the abatacept group, OPA increased after immunization with PCV (Pn6B, *p* = 0.002 and Pn23F, *p* = 0.008) but did not increase further after PPV23, and post-PPV23 OPA for Pn23F was reduced compared to controls (*p* = 0.020). In the cDMARD group, OPA increased after PCV for Pn6B (*p* = 0.017) but did not increase further after PPV23. In this group, PCV13 + PPV23 resulted in increased OPA (*p* = 0.003) for Pn23F, and post-PPV23 OPA for Pn23F was similar to controls. There were no differences between post-PCV13 and post-PPV23 OPA in the control group.

## Discussion

In this study, we found that ongoing rituximab treatment in patients with inflammatory rheumatic disease was associated with a markedly impaired antibody response to the prime-boost pneumococcal vaccination strategy. Previous studies have shown reduced immunogenicity of either single-dose PPV23 or PCV13 in RA patients with RTX [19–21], and here we observed no improvement of IgG response or opsonophagocytosis with prime-boost vaccination compared to PCV during RTX treatment. This could be expected since rituximab (anti-CD20) causes almost total depletion of B cells (> 95%) in the circulation, and B cells start replenishing 6–9 months after treatment is stopped, but these are mostly naïve or transitional B cells [22]. One year after RTX treatment, 80% of CD27+ memory B cells were still depleted and their recovery can be delayed up to 5 years [23]. Our findings strongly support the recommendation to complete pneumococcal vaccination before initiating RTX.

Prime-boost vaccination strategy resulted in positive antibody response to median 9 serotypes, compared to 7 serotypes after single-dose PCV, in IRD patients treated with conventional DMARDs, i.e., MTX, AZA, and MMF. Several groups have previously published reports of reduced immunogenicity of pneumococcal vaccines during MTX treatment [24]. When an early start of MTX treatment is necessary, i.e., in new-onset RA patients with high disease activity, the current results suggest that the prime-boost vaccination strategy is more likely to elicit a strong antibody response compared to single-dose PCV13.

Although antibody response improved with PCV13 + PPV23 compared to only PCV13 in the ABT group, the number of serotypes with putative protective IgG concentration did not increase further after PPV23. The mechanism of action for ABT is to modulate immune responses by binding to CD80/CD86 on antigen-presenting cells, thus preventing costimulatory binding of CD28 on naïve T cells and attenuating T cell activation [25]. Abatacept is known to reduce levels of switched memory B cells [26]. We hypothesize that ABT impairs the TD immune response to PCV13 with a resulting decrease in memory B cell formation and thus reducing the secondary immune response after PPV23 revaccination.

In contrast to rituximab, abatacept, and conventional DMARD treatments, glucocorticoid use (prednisolone 2.5–20 mg/day) was not associated with impaired response to prime-boost pneumococcal vaccination. Although we cannot rule out the possibility of higher glucocorticoid doses having a negative effect on antibody response, the present results are in line with our previous findings regarding single-dose PPV23 in RA patients [27] and single-dose PCV13 in patients with systemic vasculitides or RA [17, 28].

Compared to the T-independent nature of the immune response to PPV23, the TD response to pneumococcal polysaccharide-protein-conjugate vaccines not only induces opsonizing anticapsular antibodies but also results in the formation of memory cells with the potential of long-lasting immunity. The superior immunogenic properties of PCV have clear advantages over PPV23 in infants and are recommended to high-risk adults including the immunocompromised population since 2012 [15]. The sequential introduction of PCV7 and PCV13 into childhood vaccination programs worldwide has resulted in decreased incidences of vaccine-type IPD not only in infants but also in all age groups due to herd protective effects [3]. However, a shift toward IPD caused by non-vaccine serotypes, i.e., serotype replacement, has been reported from several countries [4–6]. Of all IPD cases reported to the public health agency in Sweden in 2017, only 30% were caused by serotypes included in PCV13, and the corresponding proportion in the elderly was 29% PCV13-type compared to 65% PPV23-type IPD [14]. The problem of serotype replacement has emphasized the need of a follow-up dose of PPV23 after primary PCV immunization to increase serotype coverage.

Several concerns have been raised regarding the prime-boost pneumococcal vaccination strategy. First, hyporesponsiveness following repeat pneumococcal polysaccharide antigen challenge is well established, and when PPV23 is administered after PCV7 decreased, the numbers of memory B cells have been reported [29]. Second, repeated vaccinations increase costs and compliance could be an issue. Third, in the inflammatory rheumatic disease population, vaccination timing is an important issue because necessary immunosuppressive treatment often cannot be delayed for the minimum of 8 weeks required between vaccine doses. This concern is of greatest importance in the patients with high disease activity requiring RTX treatment, as pneumococcal prime and boost immunizations have very limited immunogenicity when administered with RTX. These issues stress the need for the development of polyvalent pneumococcal conjugate vaccines, and currently a 15-valent and a 20-valent PCV are being evaluated in phase 3 clinical trials.

Our study has several limitations. Because it was not a randomized trial, there is a possibility that confounding factors have influenced statistical inferences. To counteract this risk, we performed a logistic regression analysis adjusting for possible effects of age, co-immunosuppression, and inflammatory disease activity. Although patients were older than controls, we found no association between increasing age and reduced antibody response. The heterogeneity in disease phenotypes, i.e., RA and systemic vasculitides, might have influenced antibody response. Our group has previously reported that antibody response to pneumococcal conjugate vaccine is not impaired in RA without DMARD treatment [28], but to our knowledge, there is no corresponding data regarding vaccine response in patients with systemic vasculitis without active treatment. The MFMI (Luminex) method used to measure antipneumococcal antibody is a validated method but has not been used to the same extent as the WHO standard ELISA [30]. Regardless of MFMI or ELISA, antipneumococcal serotype measurement is only a surrogate marker for protection. Different thresholds of protection have been proposed, e.g., ≥ 0.35 μg/mL for IPD in children, ≥ 1.0 μg/mL for adults, and ≥ 1.3 μg/mL for immunocompromised adults [21], but optimal correlates of protection are incompletely understood. Although the killing-type OPA assay is considered the gold standard in pneumococcal vaccine immunogenicity trials, the flow-cytometric uptake OPA assay in this study is a validated method [20], and it has previously been used in a number of pneumococcal vaccine studies [31–35]. Only two serotypes were assessed in the OPA assay, but we have no reason to believe that serotypes 6B and 23F are not representative of the immune response.

## Conclusion

Prime-boost vaccination strategy might be more beneficial compared to a single vaccine dose in patients treated with conventional DMARDs (i.e., MTX, AZA, or MMF) and also in patients receiving abatacept. Rituximab treatment lead to a large reduction in immunogenicity of single-dose pneumococcal conjugate vaccine, and a subsequent PPV23 dose did not improve antibody response. Pneumococcal vaccination should be strongly encouraged before starting rituximab.

## Data Availability

The datasets for the current study are available from the corresponding author upon reasonable request.
