# Correction to: Prime-boost vaccination strategy enhances immunogenicity compared to single pneumococcal conjugate vaccination in patients receiving conventional DMARDs, to some extent in abatacept but not in rituximab-treated patients

**DOI:** 10.1186/s13075-020-02256-2

**Published:** 2020-06-26

**Authors:** Per Nived, Göran Jönsson, Bo Settergren, Jon Einarsson, Tor Olofsson, Charlotte Sværke Jørgensen, Lillemor Skattum, Meliha C. Kapetanovic

**Affiliations:** 1 Department of Infectious Diseases, Central Hospital Kristianstad, J A Hedlunds väg 5, SE-291 85 Kristianstad, Sweden; 2 Department of Clinical Sciences Lund, Section for Rheumatology, Lund University, Lund and Skåne University Hospital, Lund, Sweden; 3 Department of Clinical Sciences Lund, Section of Infectious Diseases, Lund University and Skåne University Hospital, Lund, Sweden; 4 Department of Microbiological Diagnostics & Virology, Statens Serum Institut, Copenhagen, Denmark; 5 Department of Laboratory Medicine, Section of Microbiology, Immunology and Glycobiology, Lund, University, Lund and Clinical Immunology and Transfusion Medicine, Region Skåne, Lund, Sweden

**Correction to: Arthritis Res Ther (2020) 22:36**

**https://doi.org/10.1186/s13075-020-2124-3**

Following publication of the original article [1], the authors identified two errors of referencing in the Discussion.

In the first paragraph of the Discussion, previous references 19–21 are incorrectly used twice and should be replaced with new references 36–38 and added to the reference list as follows:

36. Bingham CO, 3rd, Looney RJ, Deodhar A, Halsey N, Greenwald M, Codding C, et al. Immunization responses in rheumatoid arthritis patients treated with rituximab: results from a controlled clinical trial. Arthritis Rheum. 2010;62(1):64–74.

37. Rehnberg M, Brisslert M, Amu S, Zendjanchi K, Hawi G, Bokarewa MI. Vaccination response to protein and carbohydrate antigens in patients with rheumatoid arthritis after rituximab treatment. Arthritis Res Ther. 2010;12(3):R111.

38. Crnkic Kapetanovic M, Saxne T, Jonsson G, Truedsson L, Geborek P. Rituximab and abatacept but not tocilizumab impair antibody response to pneumococcal conjugate vaccine in patients with rheumatoid arthritis. Arthritis Res Ther. 2013;15(5):R171.

In the fifth paragraph, reference 15 is incorrectly used twice, and should be replaced with reference 9.

The authors sincerely apologize to the readers for any confusion caused by the errors.
